# Assessment of DSM Based on Radiometric Transformation of UAV Data

**DOI:** 10.3390/s21051649

**Published:** 2021-02-27

**Authors:** Muhammad Hamid Chaudhry, Anuar Ahmad, Qudsia Gulzar, Muhammad Shahid Farid, Himan Shahabi, Nadhir Al-Ansari

**Affiliations:** 1 Department of Geoinformation, Faculty of Built Environment and Surveying, Universiti Teknologi Malaysia (UTM), Johor Bahru 81310, Malaysia; hamid.gis@pu.edu.pk (M.H.C.); anuarahmad@utm.my (A.A.); 2 Centre of GIS, University of the Punjab, Lahore 54590, Pakistan; qudsia.gis@pu.edu.pk; 3 College of Information Technology, University of the Punjab, Lahore 54590, Pakistan; shahid@pucit.edu.pk; 4 Department of Geomorphology, Faculty of Natural Resources, University of Kurdistan, Sanandaj 66177-15175, Iran; 5 Department of Zrebar Lake Environmental Research, Kurdistan Studies Institute, University of Kurdistan, Sanandaj 66177-15175, Iran; 6 Department of Civil, Environmental and Natural Resources Engineering, Lulea University of Technology, 971 87 Lulea, Sweden

**Keywords:** RAW image, UAV image transformation, UAV drone, point cloud, tie points, UAV LIDAR

## Abstract

Unmanned Aerial Vehicle (UAV) is one of the latest technologies for high spatial resolution 3D modeling of the Earth. The objectives of this study are to assess low-cost UAV data using image radiometric transformation techniques and investigate its effects on global and local accuracy of the Digital Surface Model (DSM). This research uses UAV Light Detection and Ranging (LIDAR) data from 80 m and UAV Drone data from 300 and 500 m flying height. RAW UAV images acquired from 500 m flying height are radiometrically transformed in Matrix Laboratory (MATLAB). UAV images from 300 m flying height are processed for the generation of 3D point cloud and DSM in Pix4D Mapper. UAV LIDAR data are used for the acquisition of Ground Control Points (GCP) and accuracy assessment of UAV Image data products. Accuracy of enhanced DSM with DSM generated from 300 m flight height were analyzed for point cloud number, density and distribution. Root Mean Square Error (RMSE) value of Z is enhanced from ±2.15 m to ±0.11 m. For local accuracy assessment of DSM, four different types of land covers are statistically compared with UAV LIDAR resulting in compatibility of enhancement technique with UAV LIDAR accuracy.

## 1. Introduction

Modelling Earth in X, Y and Z dimensions, generally termed as Digital Elevation Model (DEM), is becoming a compulsory data component for modelling and analysis in spatial sciences [1] which defines the variation in Z dimension of terrain digitally [2,3,4]. Any landscape or surface type described by a system of coordinates, as point elevation data on either a regular grid or triangular irregular network (TIN), or as contour strings. Such systems are collectively known as Digital Surface Models (DSM). In a further explanation, a DSM is a digital representation of the elevation in a regular grid cell structure representing Earth topographic variations with all associated natural and manmade entities. On the other hand, a Digital Terrain Model (DTM) looks like the DSM but in a way of excluding all natural and manmade objects, which are above the earth surface in order to define bare Earth. By using algorithms removing natural and manmade entities from DSM to represent only terrain resulted in DTM [5,6,7,8,9]. Data acquisition for DSM generation can be done by geodetic surveying, optical or radar satellite imagery, classical or Unmanned Aerial Vehicle (UAV) aerial photographs, and terrestrial or aerial laser scanning [10,11]. At present, DSM is one of the important and basic photogrammetric data products used in a number of applications [12]. Surveyors have been producing topographic and relief maps for many years and most modern techniques for such types of mapping are aerial photographs, stereo pairs and digital photogrammetric processes [13].

The latest medium for DSM production is aerial surveying and digital photogrammetry. Modern development in digital photogrammetric processes results in the easiest construction of DSM with very high spatial resolution and unprecedented visualizations of Earth’s surface [14]. With this modern technique, images of a scene are captured from a minimum of two different angles and the resulting overlapping images are used to create a 3D location of features in images, based upon camera focal length, position and orientation [15]. UAV technology is also termed as low-altitude remote sensing that has been used widely in many domains, and is becoming a key technology for spatial data collection in recent years [16]. UAV data products have been utilized in a wide range of remote sensing applications. In particular, the latest developments in geoinformation, computer vision and robotics have resulted in a collection of a huge amount of spatial data with low-cost UAVs. UAVs are characterized as low cost, fast and flexible spatial data acquisition systems. UAVs have a great potential for extremely high-resolution spatial data surveying and mapping tasks at low flying height [17].

The latest dissemination of remote sensing images and digital elevation data results from UAV image processing. UAV image processing techniques create a dense 3D point cloud with a structure from motion (SFM) approach developed in 1990s. Despite the fact that SFM uses the same fundamental mathematical parameters of classical photogrammetry, it is developed by image processing and computer vision community and is used as an algorithm for feature matching [18,19,20,21].

The SFM technique uses multiple overlapping images to develop a 3D surface in contrast to just two overlapping images in classical photogrammetry [22]. This technique is based on sophisticated algorithms that match randomly acquired images from multiple viewpoints and constructs the 3D model of a surface or object [23]. The standard SFM photogrammetry processing workflow is explained in [24]. The SFM process is based upon automatic triangulation of overlapping UAV imagery which is identical to the aerial triangulation process of classical photogrammetry by identification of a continuous line, polygon or identifiable points in overlapping areas of multiple images. The SFM process is generally described in three stages for DSM generation. Firstly, the estimation of relative orientation parameters (ROPs) of overlapping images by automatic identification of common point and/or line features. Secondly, by using the resulting matched points an arbitrary datum and local coordinate system are defined for estimating the image exterior orientation parameters (EOPs) and 3D coordinates of tie points. In the third stage, a bundle block adjustment algorithm is used to refine the estimated EOPs and object 3D coordinates [25]. Computer vision-based modern algorithms estimate both the interior orientation parameters (IOPs) and EOPs using the matched tie points in multiple overlapping images and the GCPs [26].

UAV technology has become a research hotspot gradually over the years with ongoing scientific and technological advancement [27]. Several investigations in the scientific literature related to hydrology, archeology, disaster management, navigation and many others are making use of UAV data. Those professionals mainly focused on computer vision-based automated workflows for DSM generation, overall accuracy assessments (Root Mean Square Error (RMSE) error calculations), number of check points (CP) used etc. Whereas the accuracy in the UAV images with reference to terrain variations has not been addressed. Most of the researchers consider that the decrease in flying height can increase the overall accuracy, but actually, it will result in more images, increased dead ground effect and also increase in computation time, which need a high-speed computer, more disk space, heavy RAM etc. Moreover, small objects, such as small holes or stones result in more outliers in extremely high spatial resolution DSM. This may influence the DSM interpretation negatively. Because of the high resolution of UAV data, mainly the researchers are not looking into the effects of spectral and/or radiometric resolution of UAV data for mapping minor topographical aspects of the earth’s surface.

UAVs are mainly equipped with cameras having a narrow field of view (FOV). This along with low flying height or low altitude results in capturing more photographs than manned aerial platforms to ensure coverage and overlaps. As a result, some images cover only homogeneous areas with less texture variation. It makes feature detection difficult. On the other hand, the higher number of images may result in a higher number of tie-points, causing more time consumption for the generation of point cloud, orthomosaic, DSM and DTM. This may also lead to short baselines and a small base-height ratio. As a result, it may cause unstable aerial triangulation and low DSM accuracy [28].

Technical advancements in spatial data acquisition and processing have contributed a great deal in enhancing mapping products’ accuracy and reducing cost, both in terms of time and labor. UAV technology as a latest spatial data acquisition technique is all contributed by robotics and computer vision developments. UAV technology is nowadays being recognized as the most cost effective for 3D modelling of the earth’s surface, but as compared to time and labor this technology is considered very high in computational cost. Low flying height image capturing with high image overlap percentage results in more images to be processed, resulting in more demand for disk space, high-end processors and high computational cost in time.

This research aims at an assessment of UAV images’ radiometric transformation as a solution for processing a smaller number of images, less storage space requirements, and less computational time which may be comparable to low flying height UAV photogrammetric products. This technique introduces a new approach by investigating the effects of radiometric transformation on relatively high altitude UAV images, which can overcome the low flying height issues discussed earlier.

## 2. Description of the Study Area

**Figure 1:**
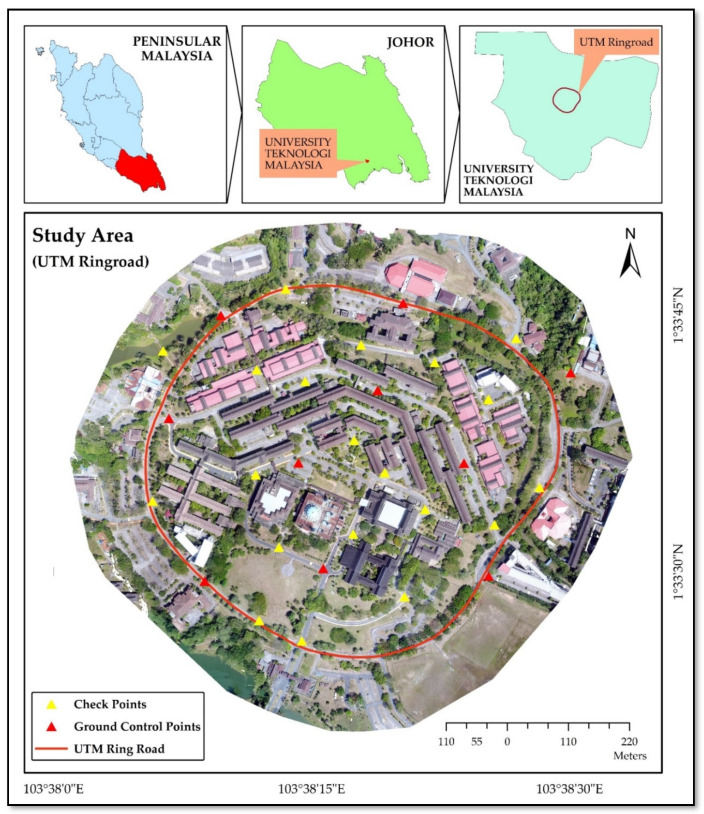
Study area with location of ground control points (GCPs) and check points (CPs).

The study site lies in the center of University Technology Malaysia (UTM), having an area of 0.51 km^2^ and shown by the red line in Figure 1. This is the main campus area of this university. This part of the university campus is built on a small hill surrounded by a ring road. That is why all the connecting roads from the campus to the ring road have a gentle slope. UTM is located in the Johor State, the most southern part of Peninsular Malaysia. This area has different manmade structures like grounds, buildings, footpaths, cafeterias and parking. Additionally, some vegetation can be found in this area. This area is a paradise for the large scale mapping experimental work because of the multiple topographic variables with a blend of modern developments.

## 3. Materials and Methods

This part is outlined two major steps data acquisition and data processing.

### 3.1. Data Acquisition

**Figure 2:**
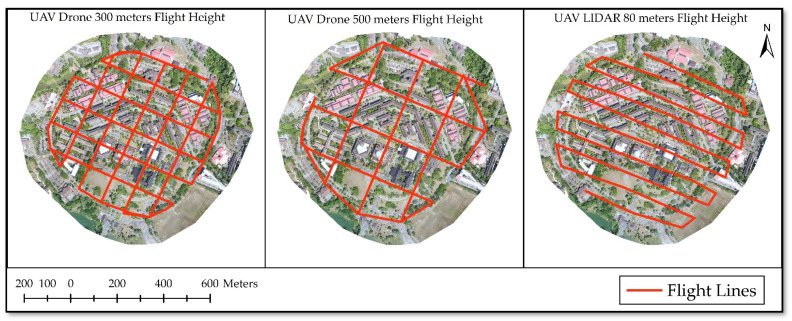
Flight Plans for 300 m (**left**), 500 m (**center**) and LIDAR 80 m (**right**).

UAV images and UAV LIDAR data were acquired for this research. UAV images are characterized by small ground coverage, huge image count, more flight lines and more data collection time [29]. Moreover, more close to the ground, the flight is more vulnerable to shadow and illumination. Therefore, this study aims at using certain image processing techniques to make high flying height UAV images usable in comparison to low flying height UAV images. For this purpose, two different flying height surveys were planned, one with 300 m flying height and the second with 500 m flying height. Figure 2 shows grid pattern flight lines of UAV used for the survey and Table 1 provides details of flight plans and their outcomes. The 300 m and 500 m flying height plans were executed in Drone Deploy software, but for the capturing of both, format .JPG and .RAW images with assistance from DJI Go 4 software were used as well. RAW data was captured because .JPG is a compressed format that may not allow further enhancement, whereas .dng is a RAW format and provides original RGB data (.dng i.e., known as digital negative).

The second category of data is UAV LIDAR data. LIDAR is an active remote sensing technology, meaning the scanner emits energy in the form of infrared laser pulses. These laser pulses are reflected off targets, which through altimetry and geolocation measurements are recorded as a point cloud that can be used to produce elevation maps [30]. LIDAR has become one of the most reliable, fast and precise techniques for the collection of topographic data [31]. LIDAR has been an optical remote sensing technique for the last two and a half decades. Air borne and terrestrial LIDAR technology are witnessing promising developments for mapping due to its high accuracy. UAV LIDAR is a relatively new technology and has been commercially available for the last few years. Thus, the application of UAV LIDAR for topographic surveying and mapping has limited research [32].

UAV equipped with a laser sensor has become popular for its capability of real time 3D data capture [33]. In this study, UAV LIDAR data is used for producing validation data layers and deriving Ground Control Points (GCPs) for UAV images registration and Check Points (CPs) for accuracy assessment of radiometric transformation processes. Table 2 depicts the detail of UAV LIDAR surveying and dataset.

*RIEGL* miniVUX-1UAV platform is used for the UAV LIDAR survey. It is a very compact UAV (RiCOPTER) integrated with a survey grade laser scanner mounted underneath the UAV platform. It weighs only 1.55 kg. Its rotating mirror which has a rotation axis along the direction of flight. It directs the laser pulses for an across-track 360° Field of View perpendicular to the direction of flight. The speed of the laser scanner is 100 scans/sec and can measure 100,000 measurements/sec. *RIEGL* miniVUX-1UAV is also mounted with two 24 mp Sony Model A600 cameras. RGB color images captured from these cameras can be overlaid with the point cloud. Data from laser sensors and cameras are managed by an on-board controller, which also includes a 220 GB SSD card sufficient for data storage of several UAV projects [34]. 

The survey is done by manually flying the UAV LIDAR from two locations because the study area has buildings so the link between drone and telemetry system may disturb it during flight. Additionally, there is a telecommunication tower located close to the study area that should be avoided since it will interfere with the frequency used by the UAV LIDAR. Ten flight lines were used to cover the whole study area as shown in Figure 2. During the survey, 1564 photos were captured by both onboard digital cameras and out of which 1233 pictures were used for the generation of orthophoto, and DSM was processed from the laser scanner point cloud directly.

### 3.2. Data Processing

In many mapping applications, high spatial and temporal resolution DSM is generated by using the latest surveying technologies such as UAV photogrammetry. In geo-sciences applications, it is mainly encouraged for its capability of surveying small areas, which is unique as compared to other techniques. Photogrammetry has been developed as the most common surveying tool because of its more sophisticated and modern computing machines [13]. At present, UAVs are being used regularly for surveying, mapping at a high spatial resolution, generating 3D point cloud, producing orthomosaic and DSM/DTM generation [35].

**Figure 3:**
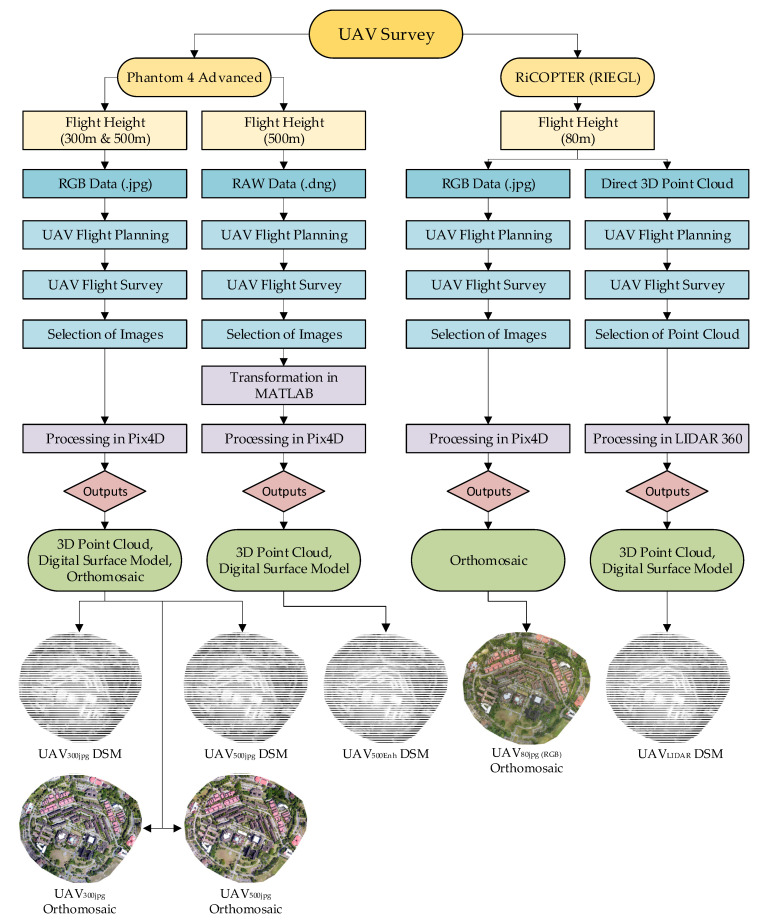
Methodology of the study.

Spatial data processing tools and algorithms have a significant effect on the quality of spatial products. As acquired datasets consist of two UAV photogrammetric projects with flying height variation and one UAV LIDAR dataset, so all three datasets were separately processed for assessment of radiometric transformation effects on DSM accuracy. Figure 3 shows a comprehensive look into the processing of three different datasets used in this study.

#### 3.2.1. UAV LIDAR Data Processing

Every new idea needs verification through some authentic technique. The accuracy of UAV LIDAR has already proved its strength in the market towards data accuracy. *RIEGL* RiCOPTER VUX-1UAV LIDAR has a built-in real time kinematic (RTK) module used for the validation survey. Table 2 shows the specifications of *RIEGL* RiCOPTER. A software RiPROCESS is provided by RIEGL along the platform which converts the scan data into point clouds. For point cloud generation, GNSS base station data are also acquired. For the reconstruction of flight plan data from both the IMU and GNSS antenna are used in GNSS base station data processing.

Laser scanner RAW data from mini VUX-1UAV were processed by using *RIEGL* RiPROCESS software, and after being colorized, the data were transferred into *RIEGL* RiSCAN PRO for filtering and contour generation. Meanwhile, digital images from RiPROCESS were exported with a geotag and further processed in Pix4D Mapper for the generation of Point cloud, DSM and orthophoto. The UAV LIDAR point cloud consists of 57,496,853 points with a point density of 111.34 sq. meters. In this research, the UAV LIDAR point cloud data are termed as UAV_LIDAR_. Figure 3 depicts the methodology used in this research.

#### 3.2.2. UAV Image Processing

Once the UAV flights were successfully executed, the acquired images are available in .JPG format for both 300 m and 500 m flying height and in RAW (.dng). Along each digital aerial image, its camera calibration parameters, geometric and spatial properties are available in form of Exchangeable image file (EXIF) data. Area coverage is different in UAV surveys from multiple flight heights. To make analysis consistent, all projects are clipped with same boundary of study area and extra images are discarded from all projects. The applied research in UAV image domain mainly uses .JPG format for processing DSM and DTM. However, exploring the potential of RAW images and image transformation techniques still need to be addressed. Therefore, UAV RAW (.dng) images captured from 500 m flying height were used for radiometric transformation, whereas UAV .JPG images captured from 300 m flying height and 500 m flying height were used for comparative analysis with radiometrically transformed products.

The 300 m .JPG images, now termed as UAV_300.jpg_, were processed in Pix4Dmapper Pro version 4.5.2 software for 3D point cloud, DSM and orthomosaic generation. PiX4D incorporates the SFM procedure described in [24,36]. For accurate DSM generation and orthophoto maps, UAV camera’s Interior Orientation Parameters (internal sensor characteristics such as focal length, principle point and camera-specific distortions) and images’ Exterior Orientation Parameters (position and orientation of the platform at the time of image capturing) are fundamental. During UAV surveying, camera location and platform position is continuously changed, resulting in change of EOPs of each image. This information can be estimated on the basis of the initial step of SFM, that is image mosaicking based upon the identification of common features in overlapped areas. This estimated information is further refined upon the basis of onboard GNSS and GCPs. Georeferencing, either direct or indirect, provides information about Exterior Orientation Parameters (EOPs). The EOPs information stored in External Information File (EXIF), which is a type of geotagged metadata along each individual image, is based upon onboard GNSS [37]. In this research, UAV images are captured by using DJI Phantom 4 Advanced. This is a low-cost, light weight UAV (1.38 kg), it has a built-in GNSS receiver, INS, and 20 MP digital camera with a 2.63 µm sensor and captures images with 4864 × 3648 pixels [38].

Algorithms in SFM procedure detect and extract local features in each image termed image feature points and afterwards match those 2D feature points throughout the overlapping images. This results in a number of tie points for potential correspondences. Tie points are those 3D points which are automatically identified and matched in the overlapping images, then used to compute from image space to object space. These tie points are used for initial image mosaicking without having information of these points in ground coordinates. Tie points and GCPs are then used in the bundle block adjustment (BBA) process to calculate a sparse point cloud in real world coordinates [39].

These are the points in image space corresponding to the similar ground points [40]. In tie points generation, the surface texture is the most important factor. Indeed, the tie point density on surfaces with a homogeneous texture (for example, water, fresh snow, and certain types of vegetation) may be low or even zero, whereas it may be extremely high in texture-rich environments such as built-up areas [41]. Indirect georeferencing gives more accurate position information as compared to direct georeferencing [32], but its accuracy depends upon the accuracy of the geodetic datum used of GCPs coordinates [42,43]. The deploying and surveying of GCPs is time consuming and can be challenging in the case of disasters or unreachable areas [44,45]. Existing elevation data sources, such as from UAV_LIDAR_, can be used for indirect georeferencing of UAV images as an alternative to deployment and surveying of GCPs [46,47,48,49,50]. The use of “LIDAR-derived control points” is termed as virtual or non-physical control points [51]. As these points are not identified on the earth surface before UAV surveying by spreading sheets on the ground, rather these points are the most identifiable marks on the earth surface, which are also easily recognizable on a large scale dataset such as LIDAR data-based orthomosaic.

In this research, UAV LIDAR-driven 10 points were used as ground control points (GCPs) for indirect georeferencing of UAV images and 18 points as check points (CPs) were used for data validation (Figure 1). The UAV LIDAR system was comprised of a *RIEGL* miniVUX1UAV LIDAR sensor, integrated with an Applanix APX-20 inertial measurement unit (IMU). The APX-20 provides positional accuracy of <0.05 m in the horizontal and <0.1 m in the vertical dimension [52].

The UAV LIDAR platform was also equipped with cameras capturing images during surveys. These UAV overlapping images were processed in Pix4D to generate orthomosaic of the study area at 3.2 cm spatial resolution. At first, the most visible and well distributed points on UAV_LIDAR_-based orthomosaic were manually identified and digitized by using ArcMap 10.3 and a shape file was created. Then, these points were overlaid on UAV_LIDAR_ point cloud and only those points from the manually digitized file were selected, which are overlaying the points in the UAV_LIDAR_ point cloud. Finally, out of all those points, 28 well distributed points were selected for further use as reference GCPs and reference CPs. This point file is used for UAV images georeferencing to generate a dense point cloud. For metric exploitation of photogrammetric data generally georeferencing is being prerequisite [45]. Once the absolute position of the 3D point cloud is known, more matched pixels can be identified in images for the generation of a 3D dense point cloud. This process is known as dense matching. This dense 3D point cloud is then manually filtered from outliers and a mesh is processed to generate DSM and orthomosaic with this UAV 3D point cloud.

#### 3.2.3. UAV Image Enhancement

UAV 500 m RAW (.dng) files are used for image processing and radiometric enhancement. Image processing is a major research area. On different research areas, scientists are working on projects such as image compression, image restoration, image segmentation etc. to enhance the existing image processing techniques and invent new methods of solving image processing problems. One of the image processing technique used in different domains effectively is the transformation of images from RGB image to grayscale [53]. In this research, UAV 500 m RAW (.dng) format was read in MATLAB as R, G and B matrices, and further used to calculate pan and grey images by using two different algorithms. In the first algorithm, TIFF images were produced by averaging spectral values of all three bands with equal weight (Equation (1)). For this research, this new single band image will be termed as UAV_pan_, calculated as:

(1) UAVpan = (R + G + B)/3

In the second algorithm, TIFF images were produced by averaging the spectral values of all three bands with specified weight (Equation (2)). The weight of bands was specified as per the standards of ITU-R BT.601-7 in which Red is 0.298%, Green is 0.587% and Blue is 0.114% [54]. For this research, this new single band image will be termed as UAV_grey_, calculated as:

(2) UAVgrey = (0.298∗ R) + (0.587∗ G) + (0.114∗B)

While running both the equations on each RAW image, the original EXIF information remained intact with every single image. Then, these images were further processed as two separate projects in Pix4D mapper software for the generation of the point cloud, orthomosaic and DSM. UAV 500 m .JPG flying height data were also processed in Pix4D mapper software but with normal processing steps to generate all the above said outputs. For georeferencing previously discussed, UAV_LIDAR_ based GCPs were also used in all projects in Pix4D mapper.

#### 3.2.4. UAV Point Cloud Filtration

**Figure 4:**
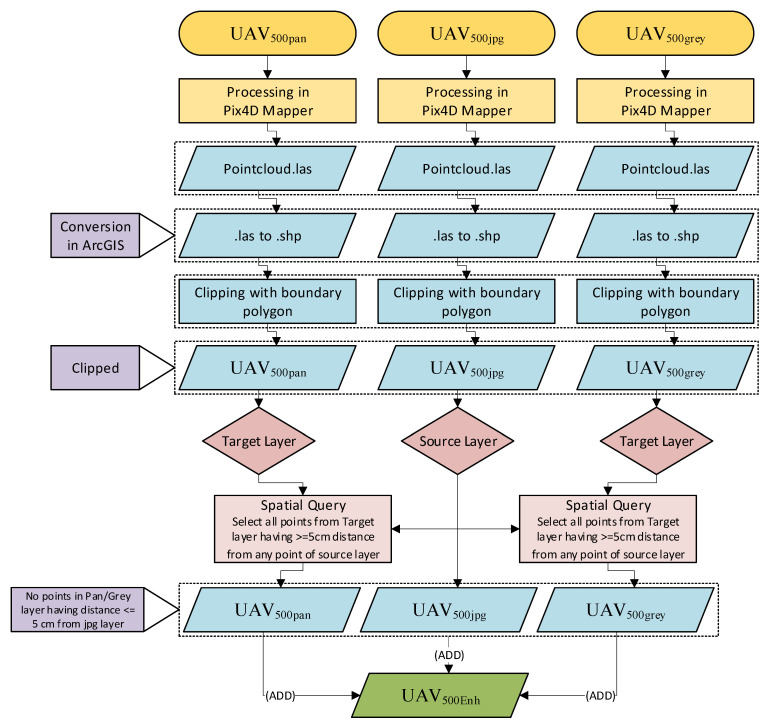
Point cloud merging and filtration.

The first product after data processing is a dense 3D point cloud based upon image matching and georeferencing of images in SFM workflow. A 3D point cloud consists of millions of points having Northing, Easting and Height values for each point. This 3D point cloud is used for further mesh building, orthophotos and DSM/DTM generation. Properties of a 3D point cloud are mainly analyzed in terms of its accuracy, completeness and distribution. Table 3 provides a comparison of the resultant changes in point clouds of multiple datasets. The objective of this study is to assess the accuracy enhancement of topographic products of high flying height UAV images comparable with low flying height UAV images. Therefore, all three dense point cloud products (UAV_500.jpg_, UAV_500pan_ and UAV_500grey_) were merged to increase the density of the point cloud. At the first step, UAV_500.jpg_ and UAV_pan_ filtered and merged on the condition that each point must lie at greater than 5 cm distance from each point of UAV_500.jpg_. For this, a spatial location query, namely “are within a distance of source layer feature” was used, the source layer in this query was UAV_500.jpg_ and UAV_500pan_ was the target layer. A similar iteration was applied to UAV_500.jpg_ and UAV_grey._ In the result, a point cloud file was generated having all points from UAV_500.jpg_ + selected points from UAV_500pan_ + selected points from UAV_500grey_. This final point cloud file has 10,152,253 usable point cloud with 19.66 m^2^ density, instead of 12,212,883 point cloud with 23.66 m^2^ density. The single dataset in terms of dense point cloud produced will now be termed as UAV_500Enh_. Figure 4 provides the processes followed for point cloud merging and filtration.

In SFM workflow, 3D dense point cloud number determines the accuracy of UAV data products. More matching points with homogeneous distribution across images results in increased accuracy of mesh generation and DSM production. As a result of this methodology, the number of point and point density in UAV_500Enh_ is more than UAV_300.jpg_. This UAV_500Enh_ point cloud generated from a lower number of images and without the geometric problems of low flying height is further used for DSM generation with accuracy comparable to UAV_300.jpg_-based DSM.

## 4. Results and Analysis

Data capturing for research by using UAV is not a new idea but there is always a room for development. UAV systems are portable and flexible technology for the acquisition of high spatial resolution aerial photographs, but still it needs geomatics and computer vision approaches for DSM generation [51,55]. The main objective of this study is the introduction of high flying height UAV images in place of low altitude flying height UAV images compared to UAV-based topographic products in terms of accuracy. To evaluate the effects of radiometric enhancement with respect to topographic variations, multiple DSM are compared in two ways: overall accuracy (global) of DSM accuracy and a local comparison of DSM accuracy with respect to topographic variations. Therefore, a comparative analysis of all products, namely UAV_300.jpg_, UAV_500.jpg_, UAV_Enh_ and UAV_LIDAR_, are given in subsequent sections.

To analyze the global accuracy of DSM, 18 CPs were used. These CPs were identified on all the observed orthomosaic for the reading of Northing and Easting information, and for the elevation value, DSM were used. There were no pre-marked GCPs, and CPs were used. CPs were selected on the basis of their identification in all orthomosaic (UAV_300.jpg_, UAV_500.jpg_, UAV_Enh,_ UAV_pan,_ UAV_grey_ and UAV_LIDAR)_ and digitized in Arc Map. Then, in Arc Map, for each CP its easting and northing values were extracted from orthomosaic and height values were extracted from DSM. The accuracy of all these CPs is computed by calculating the difference from UAV_LIDAR_ dataset values. However, while selecting the CPs, topographic variations were given due consideration (Figure 1). Table 4 depicts RMSE error calculation for CPs in multiple data sets. As for DSM accuracy assessment, height dimension is the most important. Additionally, the RMSE value of height dimension in both UAV_300.jp_ and UAV_Enh_ is ±0.11 m and as compared to similar height compressed data format (UAV_500.jpg_) the accuracy of DSM is enhanced by ±0.04 m. The results indicate similar accuracy measures of high flying height and low flying height products because of radiometric transformations.

**Figure 5:**
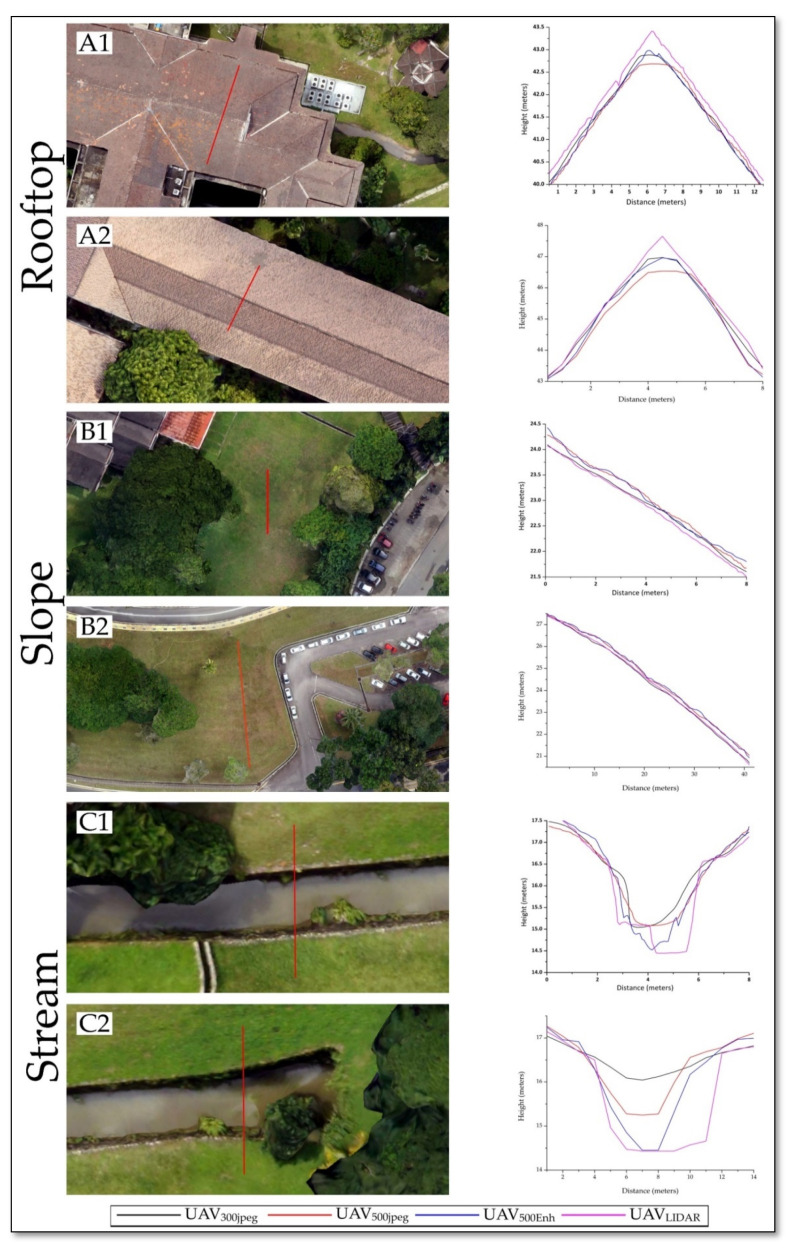
Cross-section locations: (**A1**,**A2**) Building Rooftop, (**B1**,**B2**) Surface Slope and (**C1**,**C2**) Stream. Graphs showing statistical comparison of DSM Accuracy.

**Figure 6:**
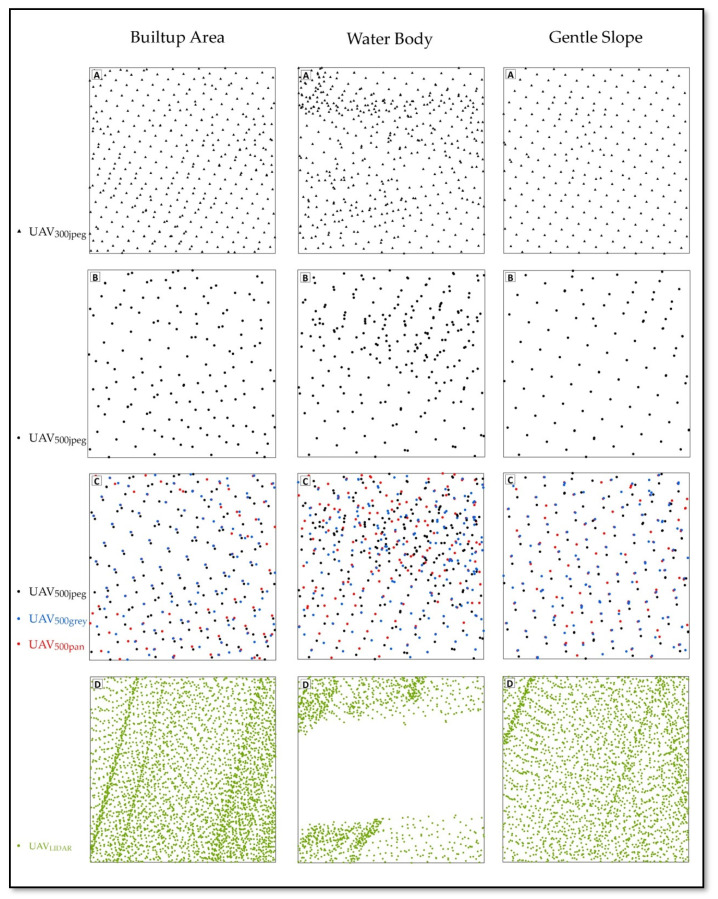
Point cloud distribution at certain area. (**A**) Showing UAV_300jpeg_ point cloud, (**B**) Showing UAV_500jpeg_ point cloud, (**C**) Showing increased number of point cloud after image enhancement UAV_500jpeg_ (black points), UAV_500grey_ (blue points) and UAV_500pan_ (red points) and (**D**) Showing UAV_LIDAR_ point cloud.

The lack of standard reporting protocol for SFM accuracy assessments and unique challenges associated with modelling different landscapes necessitates an independent evaluation of UAV survey designs for different landscapes [56]. A statistical accuracy assessment of image radiometric enhancement effects was made through cross sectional analysis of multiple DSMs, the locations of the cross sections are shown in Figure 5. In total, 16 cross sections have been evaluated in the data, and out of them the result of six cross sections are shown in Figure 6, which clearly highlights the effect of the proposed model on topographic variants. Amongst the six cross sections given in Figure 6, cross section A1 and A2 represents building rooftop, cross section B1 and B2 represents gentle slope on a grassy surface and cross section C1 and C2 represents stream. The choice of more than one cross section in similar surfaces is strongly advocating for the significance of the proposed image enhancement methodology.

In Figure 5, part A1, UAV_Enh_ is closely representing the slope variations of UAV_LIDAR_ and giving a more accurate height value as compared to UAV_300.jpg_ and UAV_500.jpg_. In part A2, enhancement technique results are comparable in UAV_300.jpg_ and UAV_Enh_, similarly B1 and B2 have normal behavior in terms of the identification of slope pattern as compared to all other data. In C1 and C2, the minimum height value difference of UAV_Enh_ is up to 50 cms from UAV_300.jpg_ and UAV_500.jpg_, and very close to the UAV_LIDAR_ data as well. LIDAR waves cannot penetrate water but it can give exact edges of water bodies so water level can be measured; in SFM workflow of UAV images water is difficult to identify due to its homogeneity, but with the introduction of image processing techniques in 500_ENH_ data, water level is better identifiable than with 300_jpg_ and 500_jpg_ data see Figure 5(C1,C2).

Table 5 provides insight into the effects on point cloud number and density, whereas Figure 6 provides insight into the effects on the distribution of point cloud. The number of points and point density in point clouds is calculated with the help of a 25 m^2^ box overlying the different land cover classes. Table 5 depicts that UAV_500Enh_ produces improved results as compared to UAV_300.jpg_.

## 5. Discussion

UAV technology as a type of aerial remote sensing is increasingly being used for the generation of orthophoto and DSM of relatively small areas. It is characterized by a low flying height of platform with a high overlap percentage of image capturing. It results in more images being processed and more prone to errors in the triangulation process. Conventional UAV surveying products used for image processing are camera images in .JPG format, multi-spectral images and thermal scanners images along with their EXIF information. This compressed format also reduces the amount of information to be interpreted [57]. The remote sensing community considers UAV’s .JPG data as high spatial resolution and high accuracy data to be used in automated workflows for orthophoto and DSM generation. Exploring the potential of image enhancement techniques is needed to address the issues of high computational cost, more processing time, information loss because of compressed format and errors introduced in data because of the low elevation of the image capturing platform.

This study demonstrates not only the potential of .dng format, which is a non-compressed image format, but also the inherent possibilities of image transformation, enhancement and GIS techniques. This study aims to acquire high accuracy of DSM with a lower number of photographs captured from a relatively high altitude. The accuracy of UAV-based DSM is governed by the number of tie points and 3D point cloud density, as more points with homogeneous distribution across images results in accurate triangulation and DSM generation process. The proposed methodology of this research results in an increase of 11.04 per square meter point density as compared to .JPG format and is 1.31 per square meter greater as compared to data captured from an elevation of 300 m flying height. Moreover, the RMSE value of enhanced data is increased ±0.04 m as compared to .JPG format and is equal to 300 m flying height UAV images data.

UAV technology is considered less accurate in areas with homogeneous texture such as roads and water bodies. Because the first stage in SFM process, that is the identification of matched points in images’ overlapping areas, is based upon image texture, more tie points are identified automatically in images with greater texture variation. Meanwhile in images where texture variation is low, a lower number of tie points are identified, resulting in a lower number of points in the point cloud. This technique also proves to be effective in such areas in comparison to low flying height imagery, .JPG format as well as UAV LIDAR technology. In road areas, the enhanced UAV images dataset exhibits an increase of 93.8 per square meter, an increase in point density as compared to .JPG format of similar elevation and is only 15.76 per square meter in comparison to low flight height UAV images. In the case of water bodies, the UAV-enhanced dataset shows an increase of 11.12 per square meter in point density as compared to .jpg format of the same elevation and an increase of 1.08 per square meter in comparison to low elevation data. This methodology gives notable results in local accuracy of DSM with only 52 images from 500 m flight height in comparison to 115 images from 300 m flight height.

## 6. Conclusions

For researchers using UAV images for certain applications in geo-sciences, photogrammetric processing of UAV images is of major research interest. It sets a great challenge for classical photogrammetric processing workflow. The existing automated software solutions can meet the requirements of most of the applications in earth sciences; still more efficient and intelligent solutions are needed to improve UAV photogrammetric projects for 3D modelling of earth surface. This research emphasizes the following investigation for UAV data applications in earth sciences: (1) Explore the potential of RAW UAV images for topographic mapping, (2) Propose a photogrammetric process model to utilize the potential of UAV RAW and .JPG images in earth surface modeling, (3) Proposed methodology to substitute GPS-derived GCPs in rugged terrain with high accuracy remote sensing products such as UAV LIDAR, and (4) Accuracy evaluation methods of photogrammetric processing apart from overall RMSE calculation.

UAV, along with its technological developments, has emerged as the most reliable low cost tool for spatial data collection. This study investigates the effects of photogrammetric processing of UAV RAW data on the quality of topographic information. Our objective is to identify the effects of images’ radiometric transformation techniques to make the UAV photogrammetric process cost effective. We have used the simplest algorithms in general, to evaluate the hypothesis as grey scale images are most commonly used for other image processing techniques more efficiently with less disk space and computational time. However, the major problem with this methodology is that the RAW format capturing capacity is not available in all sensors. Currently its testing was done on topographic features, however it cannot be adopted for all applications of UAV technology. In the weighted method, the weights of R, G and B can be adjusted with reference to the spectral response of multiple features in the study area.

UAV technology is not considered reliable for mapping areas with homogeneous texture, but this methodology gives a remarkable increase in the number of point cloud and point density for homogeneous surfaces. SFM workflow needs edges of the features to identify and use them as tie points and further use them to create dense point clouds. With this enhancement of images, before putting them into further processing, many new points were identified due to the different sharpness and reflectance patterns of pan and grey images. See Figure 5(C1,C2) where water surface level is identified only in UAV_LIDAR_ and UAV_ENH_.

The results of this study can be used for future work to investigate the effects of multiple image enhancement techniques on high resolution UAV data to remove the quality as well as increased area coverage of the topographic products and make them comparable to the low flight height data. Future studies can also investigate UAV images data application in water bodies, especially on the contaminated water where image enhancement can be better utilized, which has already been observed in cross section B1 and B2. This study gives results in comparison of 300 m and 500 m flying height data, but a comparison between some lower flight height data is also recommended. It is also recommended that an investigative study may be done on the comparison of the effects of DSM quality by using various GCPs acquiring methodologies.

## Figures and Tables

**Table 1:** Unmanned Aerial Vehicle (UAV) Flight Parameters.

Platform	DJI Phantom 4 Advanced	
Flight Height	500 m	300 m
Camera Model	DJI FC6310	DJI FC6310
Focal Length	9 mm	9 mm
Site	UTM Ring road	UTM Ring road
Study Area	0.52 sq. km	0.52 sq. km
Apps Used	DroneDeploy + DJI Go 4	DroneDeploy
Survey Date	26/10/19	06/10/19
Mode	Nadir	Nadir
Flight Lines (Gridded Pattern)	10	16
Survey Time	10:15 am	11:20 am
Avg. GSD	13.99 cm	8.4 cm
Front Overlap (%)	75	75
Side Overlap (%)	75	75
No of GCP’s	10	10
No of CP’s	18	18
No. of images	52	116
Image Format	.jpg + .dng	.jpg

**Table 2:** UAV LIDAR Flight Parameters.

Platform	*RIEGL* RiCOPTER
Flight Altitude	80 m
Survey Date	16 May 2019
Survey Time	09:00 am to 04:00 pm
Instrument	*RIEGL* miniVUX-1UAV
Camera (x2)	Sony Model A600 with 24 mp
Pulse Frequency (Hz)	100 kHz
Flight speed	4–5 m/s
Swath width	340 m
Image overlap	75%
Software used	*RIEGL* RiSCAN PRO

**Table 3:** Point Cloud comparison Generated.

Data Type	Number of Point Cloud Ready to Process	Point Density m^2^
UAV_300.jpg_	9,474,699	18.35
UAV_500.jpg_	4,449,449	8.62
UAV_500pan_	3,911,948	07.58
UAV_500grey_	3,851,486	07.46
UAV_500Enh_	10,152,253	19.66
UAV_LIDAR_	57,496,853	111.34

**Table 4:** Root Mean Square Error (RMSE) Results (meters).

	Easting	Northing	Height
UAV_300.jpg_	±0.27	±0.12	±0.11
UAV_500.jpg_	±0.25	±0.14	±0.15
UAV_Enh_	±0.26	±0.15	±0.11
UAV_pan_	±0.26	±0.15	±0.18
UAV_grey_	±0.26	±0.15	±0.20

**Table 5:** Point density at different Landcover classes.

	Number of Points			Point Density/Sq. Meters		
	Built up Area	Water Body	Gentle Slope	Built up Area	Water Body	Gentle Slope
UAV_300.jpg_	373	469	235	14.92	18.76	9.40
UAV_500.jpg_	153	218	103	6.12	8.72	4.12
UAV_500Enh._	315	496	266	12.60	19.84	10.64
UAV_LIDAR_	3433	1150	2546	137.32	46.00	101.84

## Data Availability

Not Applicable.
